# Using MDEFT MRI Sequences to Target the GPi in DBS Surgery

**DOI:** 10.1371/journal.pone.0137868

**Published:** 2015-09-14

**Authors:** Andreas Nowacki, Michael Fiechter, Jens Fichtner, Ines Debove, Lenard Lachenmayer, Michael Schüpbach, Markus Florian Oertel, Roland Wiest, Claudio Pollo

**Affiliations:** 1 Department of Neurosurgery, Inselspital, University Hospital Bern, and University of Bern, Bern, Switzerland; 2 Department of Neurology, Inselspital, University Hospital Bern, and University of Bern, Bern, Switzerland; 3 Institute for Diagnostic and Interventional Neuroradiology, Inselspital, University Hospital Bern, and University of Bern, Bern, Switzerland; University of Minnesota, UNITED STATES

## Abstract

**Objective:**

Recent advances in different MRI sequences have enabled direct visualization and targeting of the Globus pallidus internus (GPi) for DBS surgery. Modified Driven Equilibrium Fourier Transform (MDEFT) MRI sequences provide high spatial resolution and an excellent contrast of the basal ganglia with low distortion. In this study, we investigate if MDEFT sequences yield accurate and reliable targeting of the GPi and compare direct targeting based on MDEFT sequences with atlas-based targeting.

**Methods:**

13 consecutive patients considered for bilateral GPi-DBS for dystonia or PD were included in this study. Preoperative targeting of the GPi was performed visually based on MDEFT sequences as well as by using standard atlas coordinates. Postoperative CT imaging was performed to calculate the location of the implanted leads as well as the active electrode(s). The coordinates of both visual and atlas based targets were compared. The stereotactic coordinates of the lead and active electrode(s) were calculated and projected on the segmented GPi.

**Results:**

On MDEFT sequences the GPi was well demarcated in most patients. Compared to atlas-based planning the mean target coordinates were located significantly more posterior. Subgroup analysis showed a significant difference in the lateral coordinate between dystonia (LAT = 19.33 ± 0.90) and PD patients (LAT = 20.67 ± 1.69). Projected on the segmented preoperative GPi the active contacts of the DBS electrode in both dystonia and PD patients were located in the inferior and posterior part of the structure corresponding to the motor part of the GPi.

**Conclusions:**

MDEFT MRI sequences provide high spatial resolution and an excellent contrast enabling precise identification and direct visual targeting of the GPi. Compared to atlas-based targeting, it resulted in a significantly different mean location of our target. Furthermore, we observed a significant variability of the target among the PD and dystonia subpopulation suggesting accurate targeting for each individual patient.

## Introduction

Deep brain stimulation (DBS)is a neurosurgical technique including the insertion of electrodes that deliver electrical current to target nuclei[1]. The globus pallidus internus (GPi) has emerged as the target structure for DBS of dystonia and GPi-DBS is effective in the treatment of motor symptoms in PD patients[2,3]. Apart from a thorough selection of appropriate patients, the right timing of surgery and adequate stimulation parameters of the implanted electrodes, optimal electrode targeting and placement is a crucial step determining postoperative outcome in DBS surgery[4,5]. A few millimeters of targeting inaccuracy can lead to suboptimal placement of the electrode contacts within the desired target. In this case, higher stimulation intensity was needed to achieve the same therapeutic effect at the expanse of an increased risk of side effects affecting postoperative outcome.

In principle, there are two ways of targeting of the selected structure. In case of *indirect targeting*, the neurosurgeon selects the target based on a human brain atlas. The selected target in the atlas is referred to an internal reference of the patient i.e. the AC-PC line that can be located by ventriculography or magnetic resonance imaging (MRI). Distances are then deduced from the human atlas to fit to each patient. However, there are individual variations in coordinates of subcortical nuclei based on the AC-PC-line. Similarly, the anatomical and functional target might have a different spatial position in each individual. To improve targeting, microelectrode recording (MER) has been applied. MER allows visualization of neural activity of different brain structures at a multi-unit level or even single neuron activity. MER provides a qualitative information on the specific electrical firing of a given brain target and provides spatial refinement of the actual position of the intended target. The application of MER together with intraoperative macrostimulation allows assurance of the accurate electrode placement and compensates for intraoperative brain shift or imaging inadequacy[6].

Modern MRI techniques with improved image quality enable *direct targeting*: the surgeon selects the target by visualization of the intended target structure from the patient`s MRI of the head[7,8]. In principle, direct targeting should improve the problem of inadequacy of indirect targeting caused by inter-individual differences of human brain anatomy.

**Fig 1:**
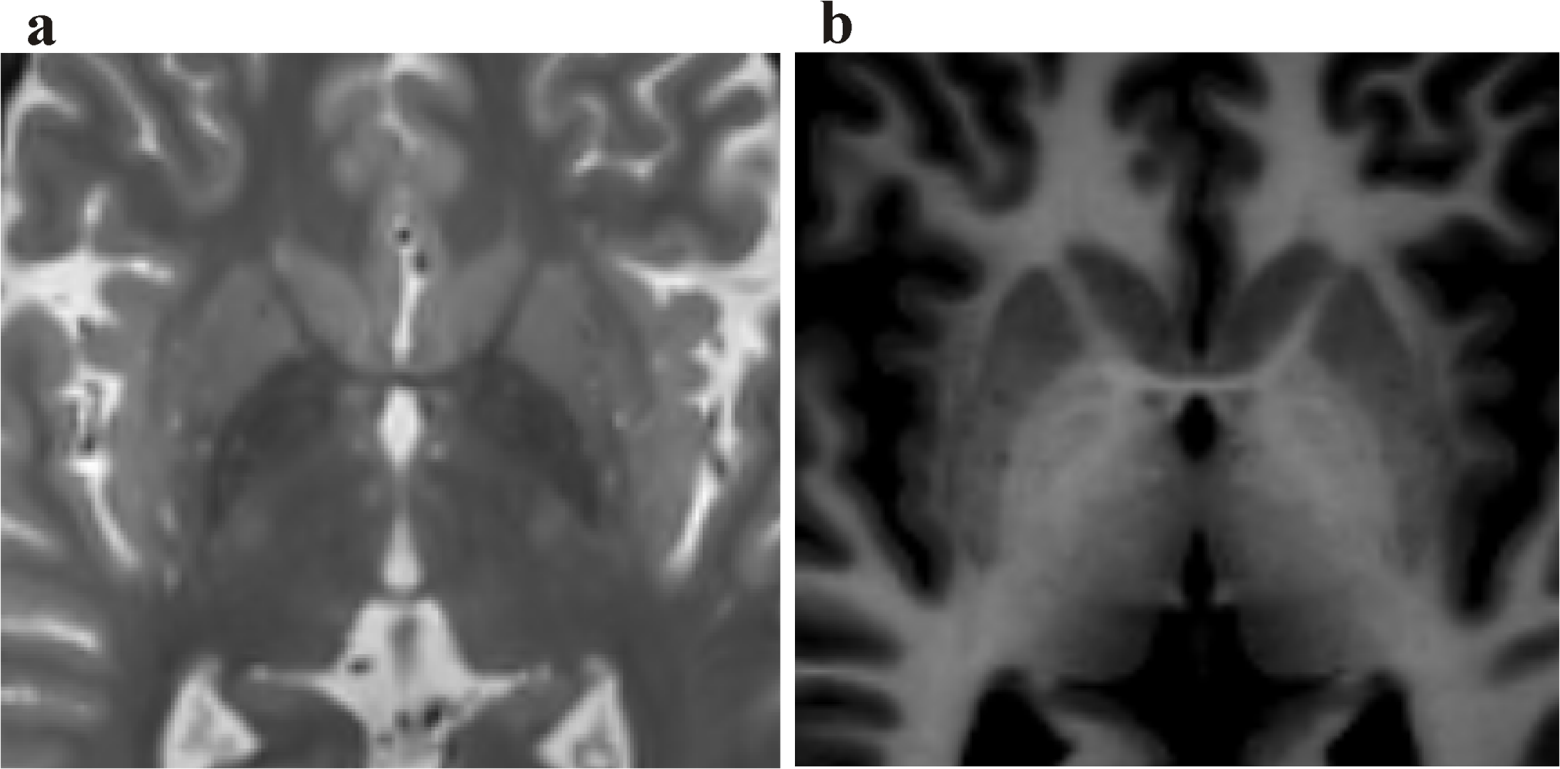
Representative axial T2-weighted (a) and MDEFT (b) sequence of a patient undergoing DBS implantation of the GPi. Note the good demarcation of the subnuclei of the basal ganglia on the MDEFT image.

Modified driven equilibrium Fourier transform (MDEFT) imaging is a *T*_*1*_-based MRI technique characterized by a high spatial resolution and an excellent contrast among grey and white matter with less gray matter density variability and a good signal to noise ratio[9]. Based on these properties, MDEFT MR imaging allows a good anatomical representation of the basal ganglia i.e. the caudate-putamen and the external (GPe) as well as internal (GPi) part of the globus pallidus (Fig 1). This qualifies MDEFT as a good candidate among MRI sequences appropriate for direct targeting in the field of DBS. The purpose of this study is to use MDEFT sequences to directly target the GPi in patients selected for DBS surgery and to evaluate the accuracy and reliability of this targeting technique.

## Patients and Methods

### Patients

In our study we prospectively analyzed a consecutive series of 13 patients with dystonia or PD undergoing bilateral electrode implantation for DBS of the GPi. Overall 26 electrodes were implanted. The population consists of 7 men and 6 women (12 adults, 1 child) with a mean age of 57,6 ± 20,1 years with PD (n = 6), inherited generalized dystonia (DYT 6, n = 1), segmental dystonia (n = 5) and inherited combined dystonia (n = 1) following the 2013 consensus classification[10]. Population characteristics are shown in Table 1. In our study, children were defined as subjects younger than 16 years of age. Extensive preoperative evaluations taking into account clinical, neuroradiological and biochemical investigations were performed by specialized movement disorder neurologists. All patients or their legal guardian provided written consent for the procedure. The study was approved by the Swiss Ethics Committee on research involving humans. All patients were clinically assessed and DBS parameters adapted and documented after three months follow-up in our outpatient clinic for movement disorders.

**Table 1:** Demographics and lead characteristics in patients with GPi DBS.

Patient	Age^a^	Sex	Disease^b^	Symptom duration (years)	Active contact position	Active contacts	Stimulation parameters [voltage (V)/ pulse width (μs)/ frequency (Hz)]
1	30	f	generalized D.	16	left: GPi	left: 0–3+	left: 4.0/ 120/ 125
					right: GPi	right: 9–10+	right: 3.4/ 120/ 125
2	51	m	segmental D.	25	left: GPi	left: c+ 1-	left: 4.0/ 60/ 130
					right: IC/GPi	right: 8–9+	right: 3.5/ 60/ 130
3	59	f	cervical D.	20	left: GPi	left: 1–2+	left: 3.2/ 120/ 125
					right: GPi	right: 9–10+	right: 3.2/ 120/ 125
4	8	m	generalized D.	7	left: GPi/GPe	left: c+ 4-	left: 2.0/ 120/ 130
					right: GPi	right: c+ 12-	right: 2.0/ 120/ 130
5	72	f	PD	14	left: GPi/GPe	left: 2–3+	left: 2.5/ 120/ 125
					right: GPi	right: 10–11+	right: 2.5/ 120/ 125
6	72	f	PD	20	left: GPi	left: c+ 3-	left: 2.7/ 70/ 130
					right: GPi	right: c+ 9-	right 2.7/ 70/ 130
7	72	m	PD	8	left: GPi	left: c+ 3-	left: 1.2/ 60/ 130
					right: GPi	right: c+ 9-	right: 1.6/ 60/ 130
8	69	m	PD	8	left: GPi/GPe	left: c+ 1-	left: 2.5/ 60/ 130
					right: GPi	right: c+ 9-	right: 2.5/ 60/ 130
9	62	m	PD	6	left: GPi	left: c+ 3-	left: 2.2/ 60/ 130
					right: GPi	right: c+ 9-	right: 2.2/ 60/ 130
10	62	m	segmental D.	36	left: GPi/GPe	left: c+ 0-	left: 4.0/ 90/ 130
					right: GPi/GPe	right: c+ 8-	right: 3.0/ 90/ 130
11	76	f	segmental D.	11	left: GPi	left: 1–2+	left: 4.0/ 180/ 125
					right: GPi	right: 9–10+	right: 4.0/ 210/ 125
12	73	m	PD	14	left: GPi	left: c+ 0-	left: 3.0/ 60/ 125
					right: IC/GPi	right: c+ 8-	right: 3.0/ 60/ 125
13	43	f	segmental D.	15	left: GPi	left: c+ 0-	left: 3.5/ 120/ 125
					right: GPi	right: c+ 8-	right: 3.5/ 120/ 125

^a^age at operation in years

^b^symptom duration in years; m, male; f, female; D, dystonia; PD, Parinson’s disease;GPi, globus pallidus internus; GPe, globus pallidus externus; IC, internal capsule, c pulse generator as anode

### MRI

Each patient received a preoperative MRI. Imaging was performed with a 12-channel head coil for signal reception on a 3 T MRI system (MAGNETOM Trio^™^ Tim, Siemens, Germany). A standard gadolinium-enhanced T_1_-weighted magnetization prepared rapid gradient-echo (MPRAGE) protocol (160 slices, 1mm thickness) was followed by 3D MDEFT sequence.

According to Deichmann et al.[9] MDEFTsequence is characterized by preparation–acquisition cycles with the structure: 90°–τ1–180°–τ2–acquisition. During the preparation part, the longitudinal magnetization is saturated by the 90° pulse, relaxes partially during τ1, is inverted by the 180° pulse, and relaxes again during τ2, resulting in a T1-weighted longitudinal magnetization. τ1 and τ2 are given by Eq 1:

τ1=quot·TIandτ2=TI−τ1 (1)

where TI is the total duration of the magnetization preparation experiment[9]. In this case, the optimized acquisition parameters included 256 x 224 x 176 matrix points yielding a nominal isotropic resolution of 1mm (repetition time 7.92 ms; echo time 2.48 ms; flip angle 16 degrees; inversion with symmetric timing 910 ms; fat saturation) and 12 minutes total acquisition time as described previously[11].

### Target planning and lead implantation

MRI was performed prior to DBS implantation. Targets and trajectories were planned based on the MRI using BrainLab software iPlan NET (Brainlab AG, Germany). The MPRAGE and MDEFT sequences were fused by applying a rigid fusion-algorithm. The neurosurgeon marked the anterior and posterior commissure (AC-PC). In a first step, the target was chosen on MPRAGE images based on the atlas of Schaltenbrand and Wahren[12] as described previously[8]. The standard coordinates were the following: lateral distance LAT_Sch_ = 20–22 mm depending on each patient`s width of the third ventricle; anterior-posterior distance AP_Sch_ = 2–3 mm depending on each patient`s AC-PC-length (2 mm if AC-PC was < 25 mm) and vertical coordinate VERT_Sch_ = 1–2 mm inferior. These atlas-based coordinates were documented in each patient. All coordinates are relative to the midcommissural point (MCP).

In a second step, planning was made independently and exclusively based on MDEFT sequences. The target was selected by visual recognition of the GPI boundaries on MDEFT sequence principally on axial as well as coronal sections. The GPI was segmented according to its demarcated boundaries (BrainLab, Germany) and the target was chosen in the posterior third of the ventral portion of the GPi maintaining a distance to the internal capsule of approximately 3 mm and to the optic tract of approximately 2–3 mm. The AP, LAT and VERT coordinates with reference to the MCP were calculated and compared to the atlas-based target coordinates.

Differences between coordinates were calculated in the AP, LAT and VERT axis as well as the Euclidian difference to compare both targets. AP, LAT and VERT distances as well as the Euclidian distance of each target related to the closest actual electrode were also calculated.

The target defined on MDEFT MRI images was used for the DBS lead implantation exclusively. The entry point was selected 2.5 to 3 cm lateral to the midline at the level of the coronal suture. Each planning was performed by the senior neurosurgeon (C.P.). The trajectory was planned so as to avoid sulci, vessels and ventricles based on MPRAGE images. On the day of surgery a Leksell G frame (Elekta instruments, Sweden) was placed and a high-resolution, stereotactic CT scan was performed and co-registrated with the preoperative MRI (Brainlab AG, Germany).

Intraoperative MER using two to three microelectrode channels was performed from 10 mm above the target in 1 mm- and further 0.5 mm-steps to confirm the boundaries of the target structure. Intraoperative clinical assessment was performed by the senior neurologist (M.S.). The intraoperative trajectories (i.e. the central one) and consequently the final position of the permanent lead were exclusively based on direct targeting on MDEFT sequences. According to the best MER and macroelectrode stimulation results, the site of implantation of the definitive electrode was chosen. The DBS electrode model Activa 3389 (Medtronic, USA) bearing 4 stimulating contacts made of a platinum/iridium alloy (1.27 mm in diameter, contact 1.5 mm in length, two adjacent contacts are separated by 0.5 mm) was implanted in each patient. The lead was fixed on the cranium using Stimloc microplates (Medtronic, USA). A postoperative high-resolution CT scan of the patients head together with the Leksell frame was done immediately after the surgical procedure to rule out hemorrhage and document correct electrode positions. One day after the initial operation a pulse generator (Activa PC, Medtronic, USA) was implanted in the infraclavicular space and connected to the DBS brain lead with a subcutaneous tunneled cable.

### Lead location and GPi segmentation

Lead locations were determined using a postoperative high resolution CT scan. Postoperative CT images were fused with the preoperatively acquired MRI MPRAGE and MDEFT sequences by applying a rigid fusion algorithm. This method was demonstrated to be accurate for identifying the postoperative lead location[13]. The AC-PC based Cartesian coordinate system was used to represent two visibly determined points along the lead: the tip of the electrode artifact (most distal point of the lead visible on the postoperative CT) was measured as well as a second point in the course of the lead typically 20–30 mm above the tip of the lead. The vector between these two points constitutes the direction vector of the lead:

$$\vec{u}=\vec{TS}$$

*with* $\vec{u}$ *constituting the direction vector between T (tip of the lead) and S (second point)*

The exact location of each active contact with its corresponding AP, LAT and VERT coordinates relative to AC-PC was calculated by the vector equation of the lead based on its known and fixed geometry of the DBS lead:

$$\vec{P}=\vec{T}\;+\;k\;\times \;\vec{u}$$

*with* $\vec{P}$ *constituting the position vector of any point P along the lead and* $\vec{T}$ *is the position vector of the tip of the lead.*

**Fig 2:**
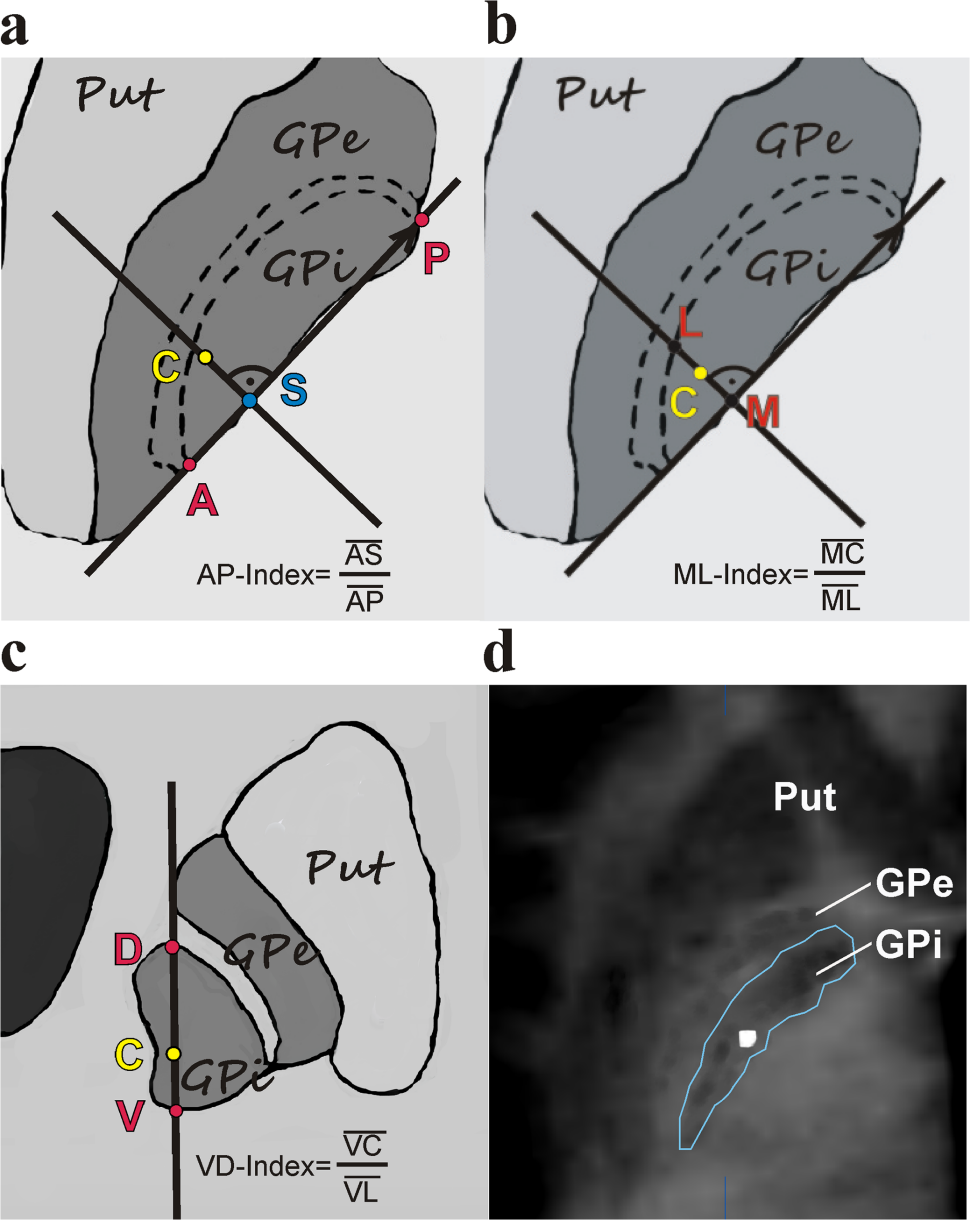
Determination of the AP-, ML- and VD-Index of the active contact C. A: A line between the most anterior (A) and posterior (P) points of the GPi constitutes the AP-line. Another line through the active contact C and perpendicular to AP intersects the AP-line in S. The AP-Index can be calculated as AP-Index = AS/AP. B: The medial (M) and lateral (L) point of the GPi are located on a line perpendicular to the AP-line crossing the active contact C. The ML-Index can be calculated as ML-Index = MC/ML. C: The ventral (V) and dorsal (D) point of the GPi are located on a line perpendicular to the ML-line crossing the active contact C. The VD-Index can be calculated as VD-Index = VC/VD. D: An overlay of the postoperative CT-scan at the level of the active contact and the MDEFT MRI sequence shows the border of the GPi after segmentation reveals the position of the active contact in relation to the anatomical borders of the GPi.

The location of the active contact was identified on the postoperative CT and projected onto the fused preoperative MRI MDEFT sequence to visualize the precise position of the active contact relative to the patient`s pallidal anatomy. Where two adjacent contacts were stimulated, the geometric mean of the two stimulated contacts was used. To determine the spatial relationship between the active contact and the GPi, we determined the segmented GPi and its anterior, posterior, medial, lateral, ventral and dorsal boundaries. The segmentation of the GPi was performed by visual depiction with the help of 3 T MDEFT sequences. Deichmann et al.[9] demonstrated excellent signal-to-noise-ratios of 37.6 ± 2.5 of 3 T MDEFT sequences allowing for reliable differentiation between grey and white matter. The posterior (P), anterior (A), lateral (L) and medial (M) points were identified visually on MDEFT sequences at the level of the active contact. P and A were determined at the posterior and anterior intersection of the internal capsule and the medial intermedullary lamina respectively. L and M were determined at the border of the GPi and the medial intermedullary lamina and the internal capsule respectively on a horizontal line perpendicular to the AP-line and crossing the active contact (Fig 2A and 2B). The ventral (V) and dorsal (D) points were set respectively at the ventral border of the GPi (transition with white matter) and the internal capsule, on a vertical line perpendicular to ML-line also crossing the active contact (Fig 2C).

The distance between each of these three axes (antero-posterior, medio-lateral and ventro-dorsal) was calculated and considered the anteroposterior (AP), mediolateral (ML) and ventrodorsal (VD) extent of the GPi. The intersection (S) of a line corresponding to each of these axes and the perpendicular of this line crossing the active contact was calculated by simple vector equation. The distance between the most anterior, medial and ventral point of the GPi and the intersection S was calculated and considered the antero-intersection- (AS-), medio-intersection- (MS-) and ventro-intersection- (VS-) distance respectively. The ratios of the AP-, ML- and VD-distances to the AS-, MS- and VS-distances were calculated (AP-Index = AS/AP; ML-Index = MS/ML; VD-Index = VS/VL) and denoted the relative distance from the active contact from the most anterior, medial and ventral border of the GPi respectively (Fig 2).

We also determined the targeting error of each lead as the AP- and LAT-distance between the intended target on pre-operative MRI scans and the actual position of the lead at the level of the intended target (same VERT value). The vector of error representing the Euclidean distance can between the intended target (AP_i_, LAT_i_) and the actual tip of the electrode (AP_t_, LAT_t_) was calculated by Eq 2:

$$d=\sqrt{{\left({AP}_{i}-{AP}_{t}\right)}^{2}+{\left({LAT}_{i}-{LAT}_{t}\right)}^{2}}$$ (2)

### Statistical analysis

Data were analyzed with descriptive/parametric statistics using SPSS software (version 20, IBM, USA). The Kolmogorov-Smirnov test was used to test for normal distribution of data sets. Two-sided student`s t-test was applied to test for statistical significance. A p-value < 0.05 was considered statistically significant.

## Results

### Target coordinates and lead location

**Fig 3:**
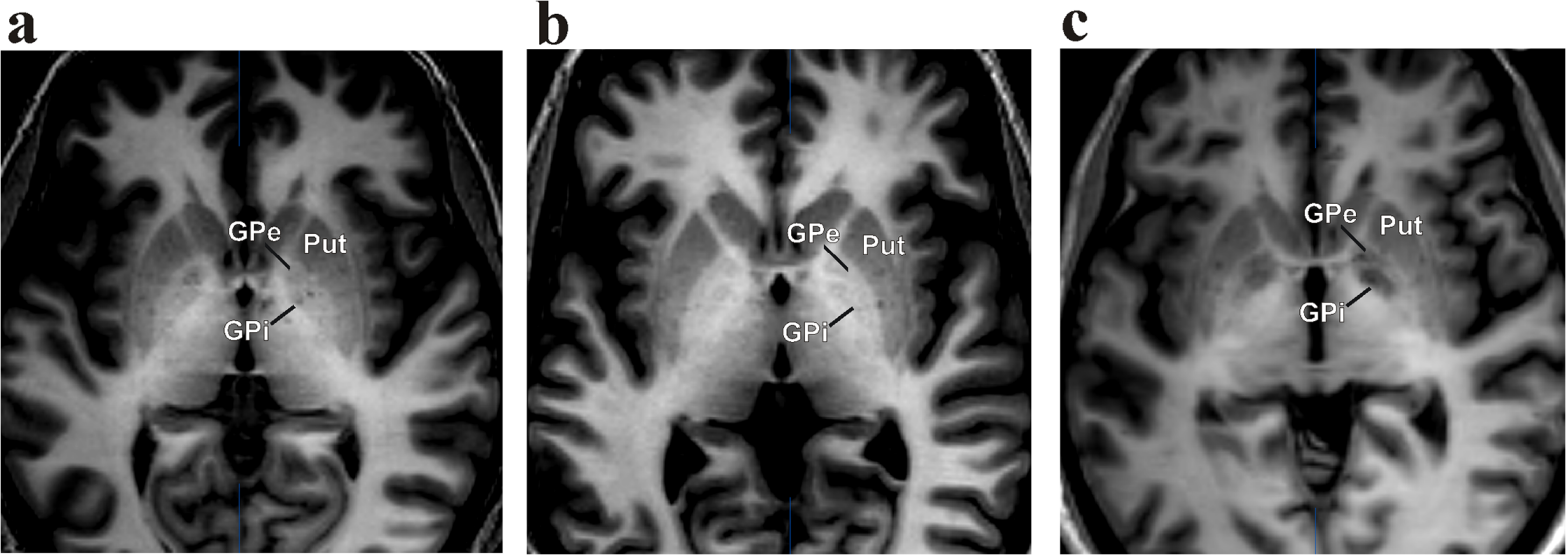
Representative MDEFT sequences of different patients undergoing DBS implantation of the GPi. (GPi, globus pallidus internus; GPe, globus pallidus externus; Put, Putamen)

The caudate-putamen and the pallidum with the subdivision of the GPe and GPi were well demarcated in most patients as the grey scale and contrast were adapted with BrainLab software for the best visualization of the target region (Fig 3).

A total of 26 atlas-based and MDEFT-based targets were obtained. We found a mean atlas-based target of LAT = 20.92 ± 0.95 mm, AP = 2,83 ± 0.34 mm, VERT = -1.91± 0.72 mm and a mean MDEFT-based target of LAT = 19.95± 1.46 mm, AP = 2,47± 0.72 mm and VERT = -2.58 ± 1.29 mm. In all three directions we obtained a statistical significance between these coordinates (Table 2). We obtained an average of differences (Δ) between target coordinates defined by MDEFT-based and atlas-based targeting of ΔLAT = 1.13 ± 0.75 mm, ΔANT = 0.61 ± 0.53 mm and ΔVERT = 1.31 ± 1.13 mm yielding a ΔEuclidian difference of 2.1 ± 1.05 mm (Table 2)

**Table 2:** Comparison of target coordinates relative to MCP for DBS leads of indirect atlas-based and direct MDEFT-based planning.

	LAT	AP	VERT	n
**atlas-based target**	20.92±0.95	2,83± 0.34	-1.91± 0.72	26
**MDEFT-based target**	19.95± 1.46	2,47± 0.72	-2.58±1.29	26
**Δ target coordinates**	1.13 ± 0.75	0.61 ± 0.53	1.31 ± 1.13	26
**p^1^-value**	0.01	0.03	0.02	

Values are means ± SD in mm

^1^ p-value calculated with unpaired t-test of same variance; n reflects the number of investigated cases.

The mean distance between the atlas-based target and closest electrode was ΔLAT = 1.04 ± 0.75 mm, ΔAP = 1.05 ± 0.62 mm and ΔVERT = 0.14 ± 0.2. Similarly, the mean distance between MDEFT-based target and closest electrode was ΔLAT = 0.8 ± 0.67 mm, ΔANT = 0.63 ± 0.4 mm and ΔVERT = 0.09 ±0.12 mm. In the LAT and AP directions, these distances were statistically significant. (Table 3).

**Table 3:** Comparison of the average of the differences (Δ) of LAT-, AP- and VERT-coordinates between the selected target defined by direct MDEFT-based and indirect atlas-based planning.

	ΔLAT	ΔAP	ΔVERT	n
**atlas-based target**	1.04 ± 0.75	1.05 ± 0.62	0.14 ± 0.2	26
**MDEFT-based target**	0.8 ± 0.67	0.63 ± 0.4	0.09 ±0.12	26
**p^1^-value**	<0.0001	0.03	0.32	

Values are means ± SD in mm

^1^ p-value calculated with unpaired t-test of same variance; n reflects the number of investigated cases.

Comparing the target coordinates of PD and dystonia patients based on direct visual targeting, a significant difference with more lateral values in the lateral coordinate in PD patients compared to dystonia patients was observed (Table 4).

**Table 4:** Comparison of target coordinates relative to MCP for DBS leads of direct MDEFT-based planning between Parkinson and dystonia patients.

	LAT	AP	VERT	n
**dystonia**	19.33±0.90	2,48±0.81	-2.32±1.36	14
**PD**	20.67±1.69	2,46±0.64	-2.89± 1.18	12
**p^1^-value**	0.03	0.93	0.27	

Values are means ± SD in mm; PD Parkinson`s disease

^1^p-value calculated with unpaired t-test of same variance; n reflects the number of investigated cases.

**Fig 4:**
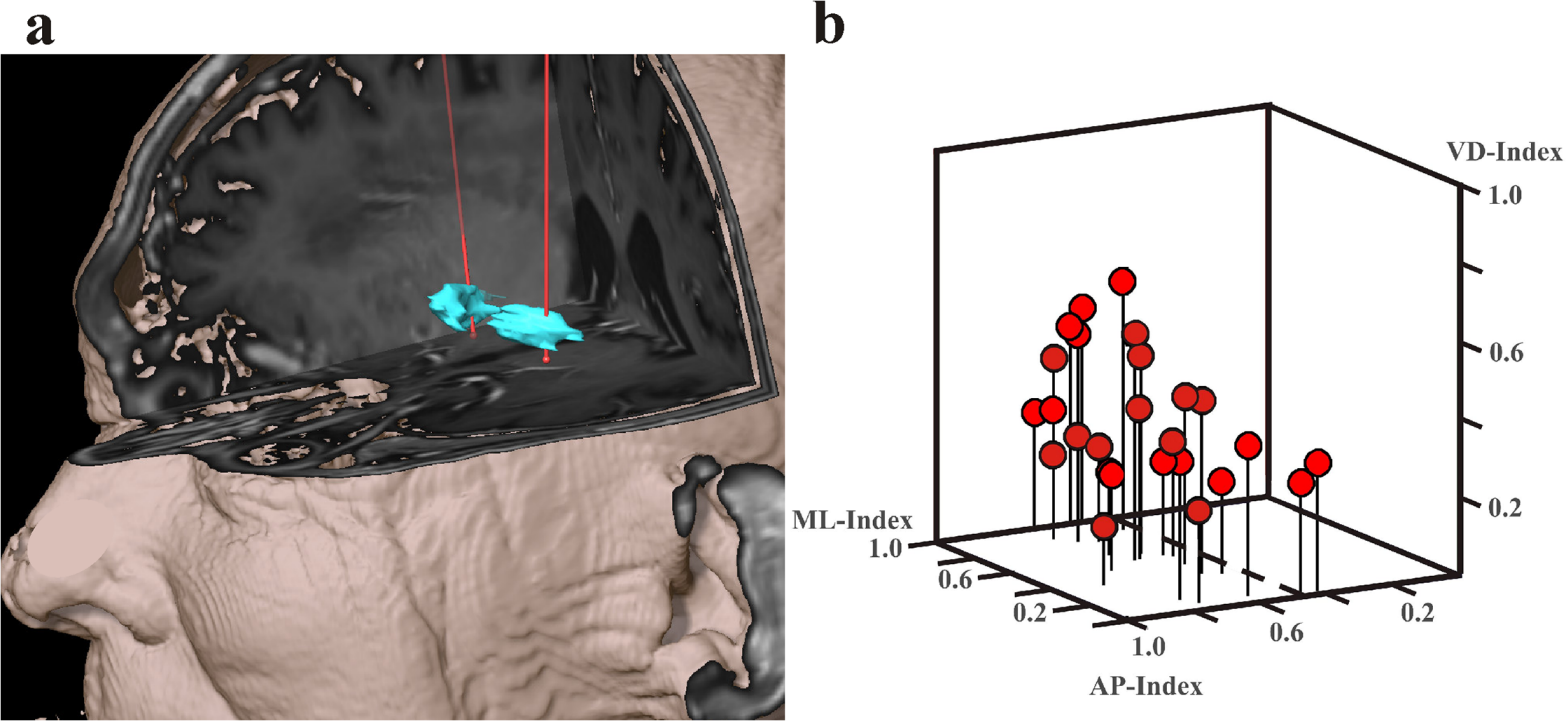
Reconstruction of the implanted DBS leads and 3D-scatterplot of the actice contact position. A: Reconstruction of DBS leads of a patient with bilateral DBS demonstrates lead location in the posterior part of the GPi. B: 3D-scatterplot of the actice contact position (red dots) relative to the border of the GPi. Position of the vast majority of the active contacts is in the posterior (AP-Index > 0.5) and ventral (VD-Index < 0.5) part of the GPi.

Across the whole cohort the mean coordinates of the tip of the lead were 19.85± 1.62mmin the lateral direction, 2.81± 1.13mmin the anterior direction and -3.77± 1.27mmin the vertical direction (all relative to MCP). The central trajectory was selected as the site of implantation of the definitive lead in 88% of all cases due to the best electrophysiological results of MER and clinical improvement after intraoperative testing. The vector of error determined at the level of the intended target was 1.07 ± 0.64 mm. There was no systematic deviation between the intended target and actual lead position. Fig 4A shows an example of the reconstruction of the implanted DBS lead crossing the GPi in the posterior part.

Further analysis of the lead location in PD and dystonia patients showed a significant difference in the lateral coordinate between these subgroups with a more lateral position of the tip of the lead in PD patients (Table 5).

**Table 5:** Comparison of the coordinates of the tip of the DBS lead relative to MCP in PD and dystonia patients with MDEFT-based planning.

	LAT	AP	VERT	n
**Dystonia**	19.13± 1.07	2.66± 1.23	-3.66 ± 1.50	14
**PD**	20.69± 1.79	2.98 ± 1.02	-3.91 ± 0.99	12
**p^1^-value**	0.02	0.46	0.61	

Values are means ± SD in mm; PD Parkinson`s disease

^1^ p-value calculated with unpaired t-test of same variance

### Location of active contacts

Across the entire cohort the mean coordinates of the stimulated contact relative to the MCP were LAT = 20.53± 1.75mm, AP = 3.89 ± 1.00mm and VERT = -0.38 ± 1.41mm. Further analysis of the active contact location between the two subgroups of PD and dystonia patients revealed a significant difference in the lateral coordinate: active contacts were located more lateral in PD patients (21.45 ± 2.00 mm) compared to dystonia (19.74± 1.01 mm) patients (p < 0.02).

When the location of the stimulating contacts relative to the internal anatomy of the pallidum was considered, we found that the active contact was located purely within the GPi in 73% of all cases. In 19% the active contact was at the border of the GPi to the GPe, in 8% of the cases it was at the border of the internal capsule and the GPi (Table 1). Further analysis of the stimulating contact relative to the borders of the GPi indicated a position in the posterior and ventral part of the GPi with a mean AP-index of 0.62, VD-index of 0.35 and a mean ML-index of 0.63 (Fig 4B). No significant differences in the AP-, MV- and VD-Indices between PD and dystonia patients were observed.

## Discussion

We justify our clinical investigation of the impact of 3 T MDEFT sequences on direct targeting of the GPi on the basis of results found in previous studies using MDEFT at ultrahigh field strength 7 T MRI. These studies demonstrated the possibility of acquiring narrow brain radiological windows near to histological resolution enabling accurate localization of brainstem therapeutic targets[14,15]. Recent results showed that MDEFT sequences yield anatomical images with high contrast and low noise levels at 3 T field strengths[9] but to date, no studies investigated the use of MDEFT for target planning in the field of DBS surgery.

Our results suggest that direct visual targeting of the GPi based on MDEFT MRI sequences is both accurate and reliable. The percentage of electrodes implanted via the central trajectory was determined as a marker of reliability. The central trajectory points to the intended target. In case of conflicting results of MER and macrostimulation combined with intraoperative clinical testing, other trajectories are tested and the permanent DBS lead is implanted according to the best MER and clinical testing results. In our series of patients the central trajectory was used in 88% of all cases, which indicates, that our intended target defined by MDEFT-based planning was set at the correct anatomical site. The mean vector of error at the level of the intended target was small with 1.07 mm indicating a close correspondence of the intended target and the actual position of the lead. Apart from targeting inaccuracy, other factors may affect the final position of the permanent lead such as (1) intraoperative brain shift induced by the guiding tube and electrode penetration into the brain, intraoperative cerebrospinal fluid loss and pneumencephalus[16,17] and/or (2) inaccuracy of the stereotactic device. Although we did not systematically analyze the different factors inducing potential brain shift, the high percentage of permanent lead implantation in the central trajectory in this series suggests a minor role of brain shift in the interpretation of our results, as we regularly and systematically check the accuracy of the stereotactic device before each surgical procedure.

Additionally, MDEFT MRI sequences enabled to perform a 3D segmentation of the GPi of each patient and to analyze the DBS lead location as well as the location of the stimulating contact with reference to the anatomical borders of the GPi. Our analysis showed that the position of the active contacts in both PD and dystonia patients was in the posterior and ventral part of the GPi. This is of particular interest as several groups have correlated the best therapeutic effect of DBS of the GPi for dystonia and PD with stimulating contact position in the posterior and ventral portion of the GPi which has been denoted the motor part of the GPi[4,18,19].

The present findings demonstrate that MDEFT MRI sequences have an impact on targeting the GPi.

Compared to atlas-based planning, the preoperative target coordinates using MDEFT sequences were located significantly more medial, inferior and more posterior. Apart from determining the differences of the average target coordinates, we also calculated the average of the differences of target coordinates, which allows us to estimate the average effect of MDEFT-based targeting technique. Determining the average of the differences of target coordinates defined by MDEFT- and atlas-based planning led to a mean targeting difference of 2.1 mm in each patient. Furthermore, we compared of the closest distance between the intended target defined by (1) MDEFT-based targeting and (2) indirect atlas-based targeting and the actual DBS lead location. Our analysis revealed significantly smaller differences of the LAT- and AP-coordinates between the intended target and actual lead location in case direct targeting, which further supports the hypothesis of MDEFT sequences leading to more accurate planning.

Interestingly, we found a difference of the lateral coordinate between PD and dystonia patients. Subgroup analysis of the preoperative target coordinates as well as the postoperative DBS lead location and the location of the active contact between dystonia and PD patients showed a significantly higher value of the lateral coordinate in the PD subgroup indicating a more lateral position of the planned target and DBS lead. The most probable explanation for this finding might be a difference in demographic characteristics between the PD and dystonia cohort as PD patients were significantly older than dystonia patients. Age-related atrophy might explain this difference[20]. In our series, the width of the third ventricle in PD patients was larger compared to dystonia patients. Apart from age there might be differences in brain anatomy due to the different underlying pathophysiological mechanisms of Parkinson disease and dystonia. Parkinson’s disease is a progressive neurodegenerative disorder whereas dystonia is not.

Furthermore, despite the difference of the mean lateral coordinate of the stimulating contact, there was no difference of the position of the active contact relative to the borders of the segmented GPi between PD and dystonia patients. This finding supports the interindividual variability of the location of the GPi, especially in these two subgroups.

Other sequences have been described for direct visualization of target structures in DBS surgery that shall be discussed below to embed our results in the context of already published work. Menuel et al. found that T2-weighted imaging resulted in greater distortions compared to T1-weighted sequences to demonstrate the subthalamic nucleus[21]. Sudhyadhom et al. described a Fast Gray Matter Acquisition T1 Inversion Recovery (FGATIR) sequence with higher contrast and contrast to noise ratio compared to MPRAGE or T2-weighted Fluid Attenuated Inverse Recovery (FLAIR) images allowing for sharper delineation of subcortical DBS target structures[22]. Although the authors gave a precise description of the qualitative (subjective visual analysis) and quantitative (contrast to noise ratio- and contrast ratio-analysis) advantages of FGATIR over other sequences, there are currently no data evaluating a potential impact of this sequence on targeting accuracy in functional neurosurgery.

Vayssiere et al. compared target coordinates of the GPi obtained by direct visual targeting based on T1-weighted MRI sequences with those determined using an atlas[8]. In accordance to our results, the authors found a significant difference between the target coordinates obtained by MRI-based and atlas-based targeting. However, no specific information about the MRI sequence properties as well as the postoperative DBS lead location is provided by the authors. Reich et al. described a 1.5 T fast spin-echo inversion-recovery (FSE-IR) sequence to target the GPi[23]. The authors found, that FSE-IR sequences lead to good signal to noise ratio but the slice thickness was relatively thick (2–3 mm).The impact of FSE-IR on targeting accuracy and postoperative outcome in DBS of the GPi was analyzed by Pinsker et al. who found that the atlas-based standard coordinates were modified in 43% of the patients based on direct visualization of the GPi[7]. The number of implanted permanent electrodes along the central trajectory was 67% and 64% respectively[24] based on IR-FSE sequences compared to 88% in our patient population. A possible explanation to this difference might be the minor slice thickness (1 mm) and higher field strength of 3 T MDEFT sequences leading to more accurate depiction of the GPi. A summary of the parameters of the different above mentioned sequences is given in Table 6.Currently, no conclusions can be drawn about which of the above discussed MRI sequences yields most accurate and reliable targeting of the GPi. Future studies comparing different MRI sequences and including clinical outcome data are needed to specifically answer this question.

**Table 6:** Parameters of different T1 weighted sequences used for targeting the GPi in DBS surgery.

	T1-w 3D MDEFT	T1-w 3D FGATIR^19^	T1-w 2D FSE-IR^21^
**Repetition time (TR)**	7.92 ms	3000 ms	3000 ms
**Echo time (TE)**	2.48 ms	4.39 ms	40 ms
**Inversion time (TI)**	910 ms	409 ms	200 ms
**Inversion pulse angle**	180°	180°	-
**Matrix**	256 x 224	320 x 256	256 x 256
**Slice thickness**	1 mm	1 mm	3 mm
**Acquisition time**	12 min	11:14 min	19 min

There are some limitations of our study. First, we performed a high resolution CT for postoperative DBS lead location and active contact position analysis. The postoperative CT-scan was co-registered with the preoperative MRI by using a rigid fusion algorithm. There is an ongoing debate about the reliability and accuracy of postoperative DBS lead location analysis based on postoperative CT and MRI. CT/MR image fusion has been demonstrated to provide an accuracy of up to 1 mm[13]. Hemm et al. analyzed the DBS lead artifact on CT and could demonstrate a precise localization of the four contact-zone of the lead[25]. Comparison of the DBS lead artifact on postoperative MRI and CT scan has been shown to be comparable and equally eligible for postoperative localization analysis[26].

Second, the sample size of our prospective study population is small and heterogeneous as it consists of PD patients and patients with various forms of dystonia. For this reason, we did not include clinical outcome data. Third, the actual DBS lead implantation was performed according to the anatomic target defined by MDEFT images exclusively. For this reason, we cannot compare leads implanted using indirect planning with leads implanted using direct planning. It would have certainly been interesting to compare leads implanted based on MDEFT sequences with leads implanted based on atlas-based targeting. However, the patients implanted according to atlas-based targeting in former times were not targeted and operated by the same surgeon and therefore we believe that it would have introduced a systematic bias when comparing these two groups. Furthermore, there is no clear reason to implant a patient using indirect atlas-based targeting with MDEFT sequences available as the sequence provides direct identification of the structure. Thus, we cannot conclude that direct targeting is superior to atlas-based targeting in terms of technical and clinical outcome. However, we could demonstrate that planning according to MDEFT leads to different mean target coordinates in the study population and a mean correction of the putative optimised target of about 2.1 mm in each individual patient.Our findings strongly suggest that direct visualization of the GPi using MDEFT MRI sequences results in accurate targeting in (1) each individual patient and (2) our two subgroups of patients. Further studies with higher patient numbers and correlation with clinical outcome have to be conducted to draw conclusions about the clinical effect of MDEFT based direct targeting of the GPi for DBS surgery.

### Conclusion

3 T MDEFT MRI sequences provide high spatial resolution and an excellent contrast among grey and white matter which enables precise identification of the GPi boundaries and direct visual targeting of the GPi for DBS surgery. Compared to atlas-based targeting, it resulted in a significantly different mean location of our target and, furthermore, a significant variability of the target among the PD and dystonia subpopulation suggesting accurate targeting for each individual patient.
